# Genicular Artery Embolization Using Imipenem/Cilastatin vs. Microsphere for Knee Osteoarthritis (GAUCHO). 12-Month Clinical Results of a Single-blind Randomized Controlled Trial

**DOI:** 10.1007/s00270-026-04504-5

**Published:** 2026-06-29

**Authors:** Mateus Picada Correa, Alexandre Froes Michelin, Lyson Azevedo Aguiar, Ricardo Lugokenski, Rodrigo Ilha Algarve, Joaquim M. Motta-Leal-Filho

**Affiliations:** 1 Instituto Vascular de Passo Fundo (Invasc), R. Capitão Araújo 297/1207, Passo Fundo, RS 99010-200 Brazil; 2 Universidade de Passo Fundo (UPF), R. Uruguai, 2001/414B, Passo Fundo, RS 99010-112 Brazil; 3 Atitus Educação, R. Gen Prestes Guimarães, 304, Passo Fundo, RS 999070-220 Brazil; 4 Hospital Ortopédico de Passo Fundo, Av. 7 de Setembro, 817, Passo Fundo, Brazil; 5 Clínica Cedil, Av. 7 de Setembro, 817, Passo Fundo, Brazil; 6 School of Medicine, Instituto Do Câncer Do Estado de São Paulo, Universidade de São Paulo (USP), Av. Dr. Arnaldo, 251, Cerqueira Cesar, São Paulo, SP 01246-000 Brazil; 7 School of Medicine, Instituto Do Coração (InCor), Universidade de São Paulo (USP), Av. Dr. Enéas de Carvalho Aguiar, 44, São Paulo, SP 05403-900 Brazil

**Keywords:** Genicular artery embolization, GAE, TAME, Knee ostheoartritis, Musculoskeletal embolization, Synovitis

## Abstract

**Purpose:**

To compare the safety and clinical outcomes of genicular artery embolization (GAE) performed with permanent microspheres (PM) and imipenem/cilastatin (*I*/*C*) in patients with mild-to-moderate knee osteoarthritis.

**Materials and Methods:**

In this IRB-approved prospective, randomized, single-center, single-blind trial, 60 patients with mild-to-moderate knee osteoarthritis were allocated to GAE with *I*/*C* (*n* = 30) or microspheres (*n* = 30). Clinical outcomes were assessed at baseline, 30 days, 90 days, and 365 days using Visual Analog Scale (VAS), *Western Ontario and McMaster Universities Osteoarthritis Index* (WOMAC), and *Knee Injury and Osteoarthritis Outcome Score* (KOOS).

**Results:**

Baseline characteristics were similar between groups, except for a higher rate of bilateral treatment in the microsphere group (56.6% vs. 30.0%, *p* = 0.037). Technical success was achieved in all procedures, with lower embolic volume in the microsphere group (*p* = 0.003). The mean volume of embolic was 1.5 ml in the microsphere group and 2.0 ml in the *I*/*C* group (*p* = 0.003). Between-group comparisons showed no significant differences in VAS at 30, 90, and 365 days (3.20 vs. 2.28, *p* = 0.102; 3.38 vs. 3.33, *p* = 0.931; and 3.95 vs. 3.88, *p* = 0.928), in WOMAC pain (6.25 vs. 4.83, *p* = 0.182; 8.11 vs. 6.00, *p* = 0.051; and 7.50 vs 6.27, *p* = 0.323), or in KOOS pain (63.59 vs. 72.12, *p* = 0.115; 56.58 vs. 67.14, *p* = 0.074; and 53.76 vs. 65.81, *p* = 0.081) for *I*/*C* versus microspheres, respectively.

**Conclusion:**

GAE using either *I*/*C* or microspheres is safe and effective, with significant clinical improvement over 12 months and no major differences in adverse events and pain outcomes between embolic agents.

**Supplementary Information:**

The online version contains supplementary material available at https://doi.org/10.1007/s00270-026-04504-5.

## Introduction

Genicular artery embolization (GAE) has emerged as an effective treatment option for patients with knee osteoarthritis (OA) who are refractory to conservative treatment but are not yet candidates for total knee arthroplasty [1]. The rationale behind GAE is to occlude pathological neovessels formed in the synovium as a result of chronic inflammation that causes pain, while preserving normal arterial circulation.

Originally, GAE was performed using imipenem/cilastatin (*I*/*C*) as an embolic agent [1]. Its use was based on its crystalline properties and a safe temporary embolic effect [2]. Experimental studies demonstrate recanalization of *I*/*C* within 48h of vessel occlusion [3] and resolution of intestinal bleeding with no ischemic complications [4]. However, *I*/*C* is not approved as an embolic agent and its off-label use for GAE is not permitted in many countries, which limits its widespread adoption.

In this setting, since the initial publication by Okuno et al., [1] permanent embolics have been increasingly used. Different cohort studies using permanent microspheres (PM) for GAE have reported similar clinical benefits to those observed with *I*/*C *[5–9]. However, permanent embolic agents may raise concerns regarding ischemic complications in musculoskeletal embolization and there has not been a prospective controlled study directly comparing *I*/*C* and microspheres. As a result, it remains unclear whether the choice of embolic agent significantly impacts the safety and efficacy of the GAE procedure. To help address this knowledge gap, a randomized controlled trial was conducted to compare the safety and efficacy of GAE using *I*/*C* versus PM to treat chronic knee pain related to OA.

## Material and Methods

### Study Design

This was a randomized, single-blind, prospective, single-center trial approved by the Ethics Committee of Universidade de Passo Fundo.

Patients with mild-to-moderate knee osteoarthritis, defined as Kellgren–Lawrence (KL) grades 1–3 and a Visual Analog Scale of pain (VAS) score ≥ 6 for more than 6 months despite physical therapy or intra-articular injection, were included in this trial. Indications for surgery, KL grade 4, secondary arthritis, and fibromyalgia were defined as exclusion criteria. Details of the study design are provided in Supplemental Table 1 and in the previously published study protocol [10]. All patients provided written informed consent.

**Table 1:** Patient demographics

	Global (*n* = 60)	*I*/*C* (*n* = 30)	Embosphere (*n* = 30)	*P*
Age, mean (SD)	63.72 (13.29)	64.37 (9.78)	63.44 (11.18)	0.729
BMI, mean (SD)	31.96 (5.02)	33.49 (5.03)	31.08 (5.51)	0.084
VAS, mean (SD)	8.13 (1.76)	8.00 (1.71)	9.00 (1.83)	0.213
Time with pain, mean (SD)	92.95 (71.19)	84 (61.30)	104 (79.27)	0.240
Sex, *N* (%)	Female: 55 (91.6). Male: 5 (8.3)	Female:27 (90). Male: 3 (10)	Female:28 (93.3). Male: 2 (6.6)	1.00
Race, *N* (%)	Caucasian: 49 (81.66). Black: 11 (18.33). Total: 60 (100)	Caucasian: 29 (96.6). Black: 1 (3.3). Total: 30 (100)	Caucasian: 20 (66.6). Black: 10 (33.3). Total: 30 (100)	0.11
Side, *N* (%)	Right: 42 (51.21). Left: 40 (48.78). Total 82 (100)	Right: 23 (57.5). Left: 19 (45.23)	Right: 17 (42.5). Left: 23 (57.5)	0.827
Bilaterality, *N* (%)	26 (43.33)	9 (30)	17 (56.66)	0.037
Kellgren–Lawrence grade, *N* (%)	1: 28 (34.14) 2: 34 (41.46) 3: 20 (24.39) Total: 82 (100)	1: 12 (28.57) 2: 19 (45.23) 3: 11 (26.19) Total: 42 (100)	1: 16 (40) 2: 16 (40) 3: 8 (20) Total: 40 (100)	0.697

*I*/*C* = imipenem/cilastatin; SDP = standard deviation; BMI = body mass index; VAS = Visual Analog Scale

### GAE Procedure and Randomization

**Fig. 1:**
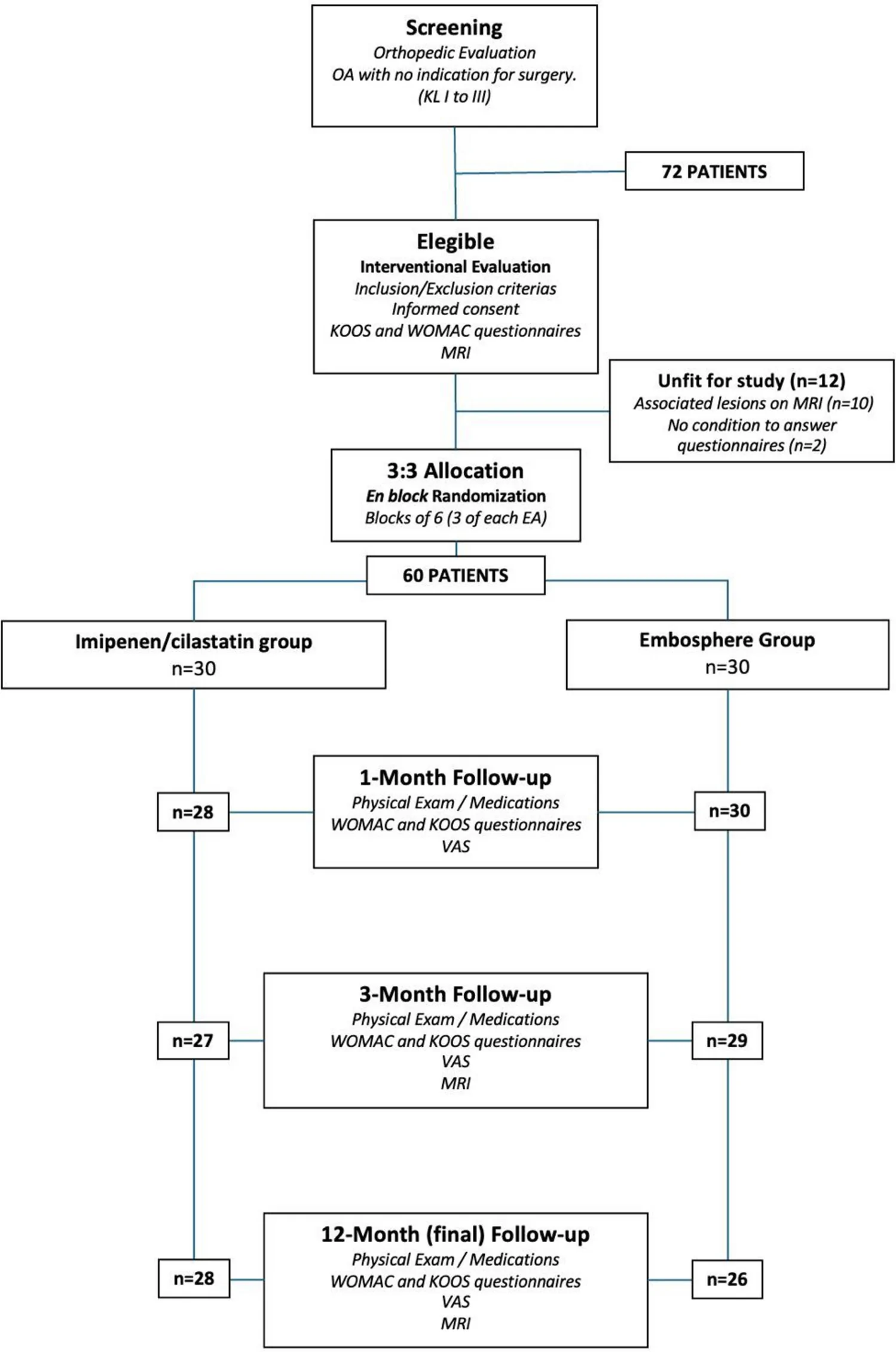
Flowchart of the study. KL, Kelgren–Lawrence classification; KOOS, Knee injury and Osteoarthritis Outcome Score; WOMAC, Western Ontario and McMaster Universities Osteoarthritis Index; MRI, magnetic resonance image; EA, embolic agent; VAS, visual analogue scale of pain; n, number of patients

An *en bloc* randomization of 60 patients was performed (Fig. 1). Randomization was performed in blocks of six, with three envelopes labeled *I*/*C* and three labeled microspheres in each block. Within each block, the envelopes were shuffled to generate a random allocation sequence, and patients were assigned sequentially according to the order of the envelopes at the time of the treatment. After completion of one block, a new block was prepared and shuffled using the same method [11, 12]. Patients were treated in the Cath Lab using an Opescope Acteno Shimadzu device (Shimadzu Corporation, Kyoto, Japan). The procedure was performed by 2 interventional radiologists with 12 and 15 years of experience in catheter-based embolic procedures and 2 years of experience in GAE. Patients were blinded to the embolic agent used.

Prior to the procedure, patients were examined in the prep room; areas of knee tenderness were identified based on the corresponding areas of synovitis on magnetic resonance imaging (MRI), and pain intensity was recorded using the VAS. If patients were indicated for bilateral treatment, both knees were treated in the same session, always starting with the right knee and then the left.

**Fig. 2:**
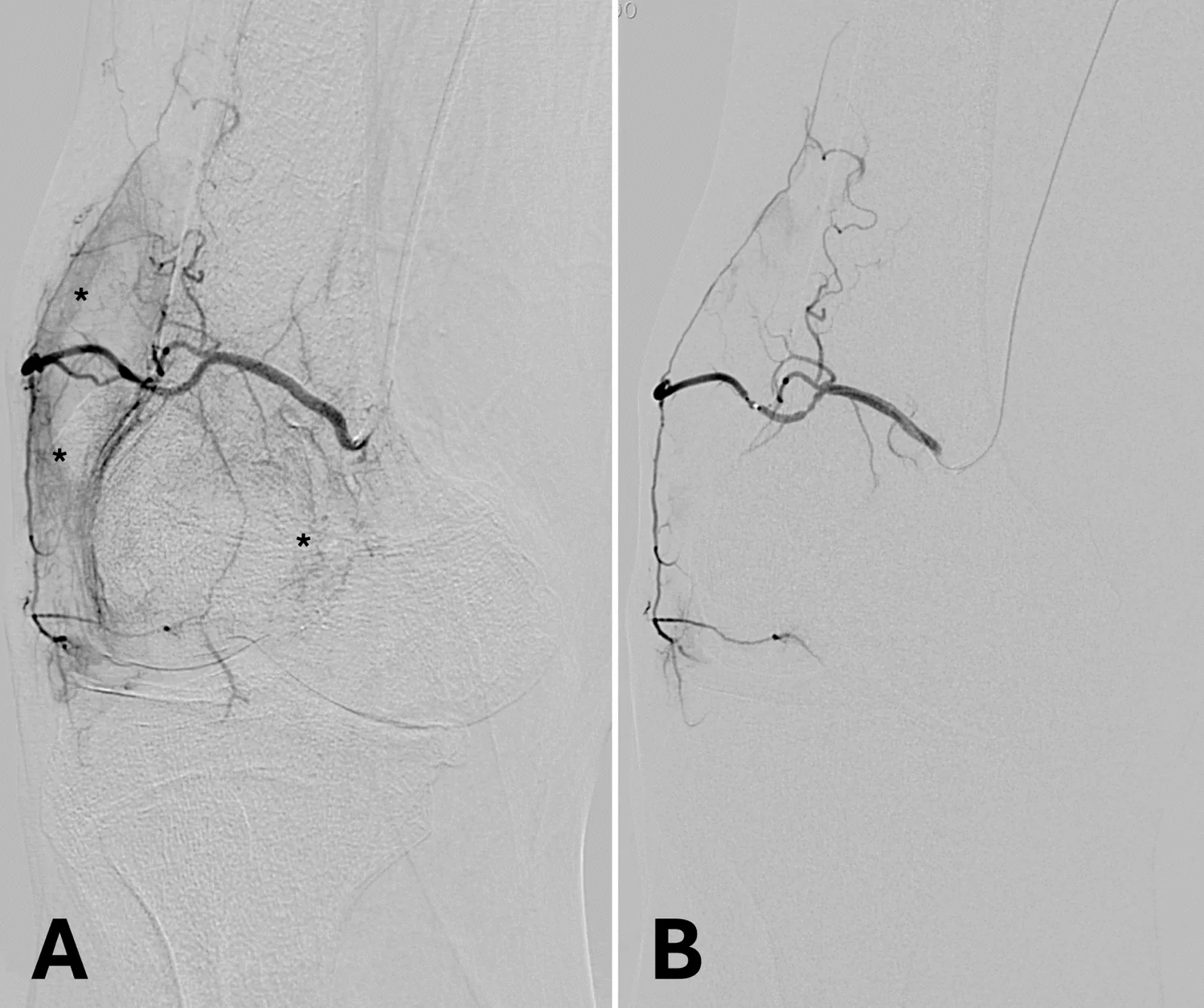
Superselective pre (**A**) and post (**B**) angiogram of the superior lateral genicular artery. After the embolization, there was no evidence of neovessels (*) in the study

Under local anesthesia without sedation, ipsilateral, anterograde, percutaneous arterial access was obtained with a 5F introducer sheath placed in the common femoral artery. Access to the superficial femoral artery was obtained with a 5F internal mammary (IM) catheter, with panoramic arteriography by manually injecting 10 mL of diluted contrast media, with a 10-mL syringe to identify the genicular arteries and the neovascularization blush. After selective catheterization of the ostia of the genicular arteries with the IM catheter, superselective catheterization with a 2.1F Maestro microcatheter and a 0.014-inch Trueform guidewire (Merit Medical Systems, South Jordan, UT, USA) was performed. Target inflammatory areas were identified as a tumor-like blush adjacent to the bones in the arterial phase of the angiogram. During superselective arteriography, the microcatheter was advanced as close to the synovial tissue as possible to precisely identify the blush. If the patient confirmed that the pain corresponded to the target area, GAE was performed using the embolic agent allocated at randomization: I/C (500 mg + 500 mg) (Biochimico, Rio de Janeiro, RJ, Brazil), 1 vial diluted in 10 mL of contrast media; or 100–300 µm Embosphere microspheres (Merit Medical Systems, South Jordan, UT, USA) diluted in contrast media, for a 20-mL total solution (2 mL of Embosphere + 9 mL of saline + 9 mL of contrast media). The technique involved injecting 0.3-mL aliquots of embolic agent with a 1-mL syringe to embolize only the blush area. The embolization was performed in at least one genicular artery corresponding to the area of pain, until the blush was eliminated and pain improved on immediate palpation, while preserving the native distal genicular branches (Fig. 2). Technical success was considered achieved with at least 1 feeding artery treated in each joint. After confirmation of pain reduction in all the target areas by physical examination in the operating room, a final angiography was performed, followed by manual compression at the puncture site, and patients remained flat for 6h before discharge.

### Efficacy

Clinical success was defined as a 50% reduction in the Western Ontario and McMaster Universities Osteoarthritis Index (WOMAC) pain scores or an increase of ≥ 10 points in the Knee Injury and Osteoarthritis Outcome Score (KOOS) pain from baseline to 3 months and 12 months post-GAE. WOMAC is a disease-specific measure for knee osteoarthritis consisting of 24 items distributed across 3 subscales: pain (0–20), stiffness (0–8), and physical function (0–68). Higher WOMAC scores indicate worse patient condition [13–15]. KOOS consists of 5 subscales designed to quantify changes in knee osteoarthritis after intervention. A normalized score is calculated for each subscale, where 100 indicates no symptoms and 0 indicates extreme symptoms [16, 17].

The immediate, intraprocedural response to GAE was evaluated using the Visual Analog Scale (VAS), a one-dimensional measure of pain intensity from 0 to 10. Clinical success for immediate pain improvement was defined as a 70% reduction in baseline VAS score at discharge compared with the baseline VAS score recorded at admission.

Technical success (i.e., superselective catheterization and embolization of at least one feeding artery supplying the hyperemic synovium of the knee joint) was assessed as well as procedural characteristics.

Secondary study measures included safety and reductions in analgesic use over the follow-up. The results obtained for KL grade 1–2 and for KL grade 3 were compared between treatment groups.

### Safety

Complications were reported according to the Classification of Complications and the Society of Interventional Radiology Adverse Event Severity Scale [18].

### Radiological Evaluation

The MRI assessment was performed by a radiologist with 16 years of experience in musculoskeletal radiology at baseline, 3 months, and 12 months. Pre-procedural MRI was used to identify the presence and distribution of synovitis, to guide target area selection during the procedure, and to exclude associated pathologies, such as tumors, fractures, or other causes of pain not related to osteoarthritis. The post-procedural MRI was used to assess potential complications (e.g., ischemia, bone infarction, osteonecrosis).

### Medications

Self-reported use of analgesics, nonsteroidal anti-inflammatory drugs (NSAIDs), and neuropathic pain medications for knee pain was recorded. The amount, frequency, and classes of medications in both groups were compared. The frequency of medication use was assessed based on the number of pills taken per week.

### Clinical Follow-up

Assessments were recorded at baseline, 30 days, 3 months, and 12 months personally by the researchers. Patients were oriented to answer the questionnaires at home earlier in the day of every visit.

### Statistical Analysis

This study was designed as a randomized non-inferiority trial. An absolute difference of 10% in the success rate in favor of permanent microspheres and a desired statistical power of 80% were considered for sample size calculation, assuming a non-inferiority margin of 20 percentage points. Using a one-sided test to assess non-inferiority, 26 patients were required in each treatment group.

Quantitative variables with normal distribution were summarized using mean and standard deviation, and asymmetric variables by the median and interquartile range. The Kolmogorov–Smirnov test was used to test for normality. The Student’s t test for independent samples or the Mann–Whitney U test was used to compare groups at baseline. Qualitative variables were described by frequencies and percentages and compared using the Chi-square test at baseline.

The results were described in a global analysis (*I*/*C* and permanent microspheres), an intragroup analysis (results of the procedure over time in each study arm), and an intergroup analysis (i.e., the comparative results between the two embolic agents). Global and intragroup results were analyzed using Generalized Estimating Equation (GEE) models with Bonferroni correction for multiple comparisons. The estimated marginal means with their respective 95% confidence intervals (CI) were used for the VAS, WOMAC, and KOOS assessments. In patients with bilateral treatment, GEE models were used to account for within-patient correlation.

All analyses were conducted using SPSS version 20.0. The threshold for significance was 0.05.

## Results

### Overview

From February 2021 to December 2022, fifty-two women and 8 men were enrolled; the mean age was 63.72 (± 13.29) (Table 1). Both groups were generally well-matched on demographics, except for a higher rate of bilateral treatment in the PE group. There were no differences observed in WOMAC and KOOS at baseline.

Eighty-two knees were treated, 75 knees (91.4%) by ipsilateral puncture, and 7 knees (8.5%) by contralateral puncture. Angiographic blush was identified in all patients. Technical success was achieved in all patients. The mean number of arteries treated per knee was 2.74 (± 0.98) with no statistical difference between the groups (*p* = 0.081). The mean injected volume of the embolic agent solution per knee was 1.93 (± 0.9) ml. The mean volume of the *I*/*C* group was 2.00 (95% CI: 1.5, 3.2) ml. In the PM group, the mean volume was 1.5 (95% CI: 1.2, 2.02) ml. The volume of embolic used was significantly less for patients treated with PM vs. *I*/*C* (1.5 ml vs. 2.0 ml; *p* = 0.003) (Table 2).

**Table 2:** Genicular arteries treated and volume of embolic agents used

	Arteries treated (N; %)			Volume of embolic agent (mL; ± SD)		
Arteries	Imipenem/cilastatin	Microspheres	*P* value	Imipenem/cilastatin	Microspheres	*P* value
Patellar	3 (7.1%)	1 (2.4%)	0.616	0.73 (0.67)	0.3	0.637
Descending	23 (54.8%)	24 (57.1%)	1.0	1.19 (0.58)	0.65 (0.32)	< 0.001
Superior lateral	16 (38.1%)	23 (54.8%)	0.189	0.73 (0.42)	0.4 (0.15)	0.003
Superior medial	18 (42.9%)	14 (33.3%)	0.501	0.86 (0.4)	0.59 (0.34)	0.053
Median	1 (2.4%)	9 (21.4%)	0.015	0.9	0.32 (0.13)	0.137
Inferior medial	22 (52.4%)	32 (76.2%)	0.04	0.87 (0.52)	0.52 (0.3)	0.004
Inferior lateral	23 (54.8%)	19 (45.2%)	0.513	0.7 (0.41)	0.79 (0.41)	0.384
Recurrent anterior tibial	2 (4.8%)	1 (2.4%)	1.0	0.6	0.6	1.0

Total number of arteries treated: 231; 8 (3.4) arteries dissected

Legend: *N* = number; mL = milliliters; SD = standard deviation

The mean procedure time was similar for both treatment arms (*I*/*C*: 24.51 ± 8.59; PM: 22.21 ± 7.24; *p* = 0.191). The mean fluoroscopy time was 10.5 ± 3.8 min, with no difference between the groups (*p* = 0.329). The median radiological exposure of the *I*/*C* group was 24.99 mGy (17.94–40.20). In the PM group, the median was 19.36 mGy (13.92–28.64), with no difference between groups (*p* = 0.162).

### Efficacy

**Fig. 3:**
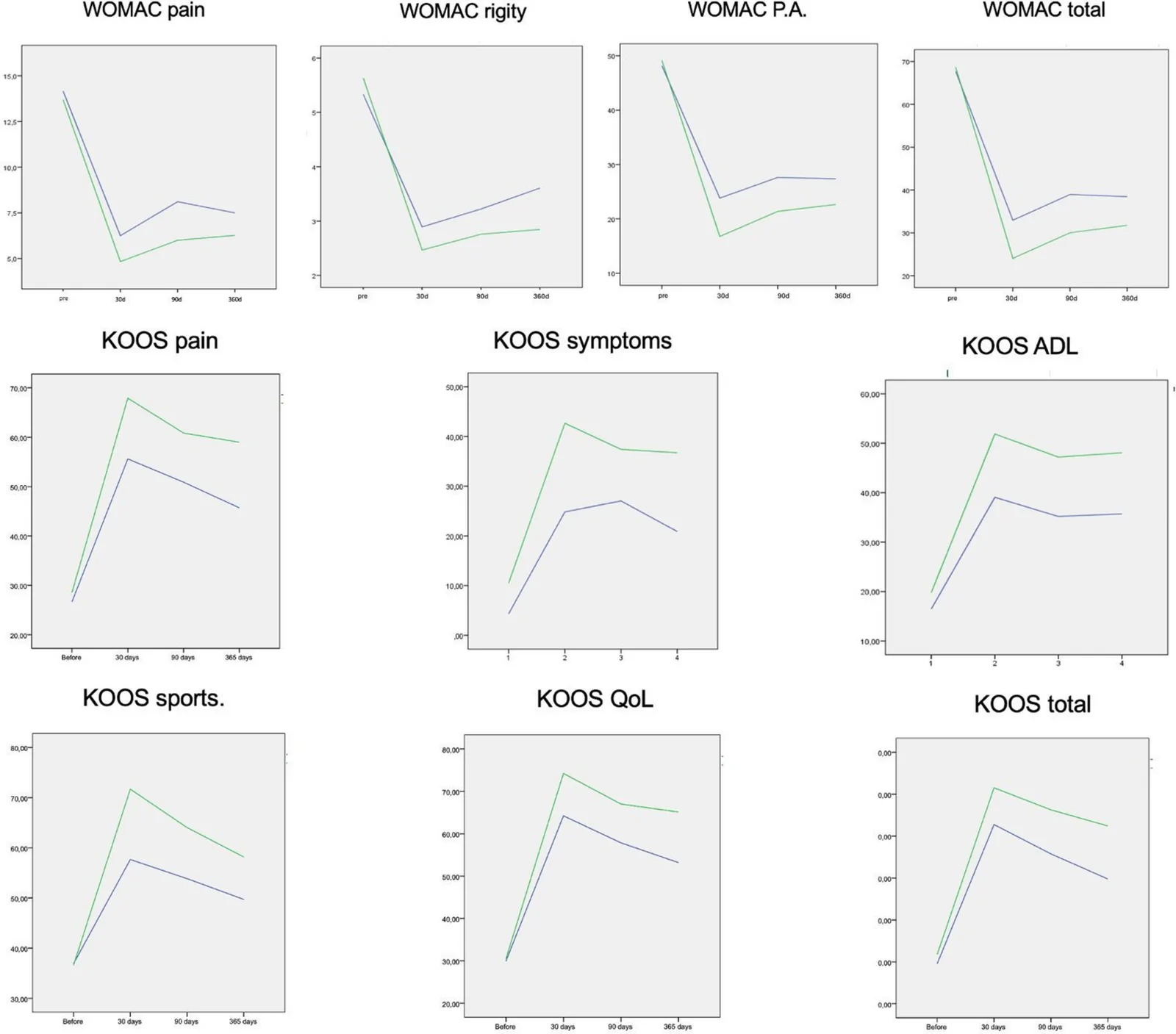
Results of Estimated marginal mean over time of imipenen/cilastatin (blue line) and microsphere (green line), in the WOMAC (Western Ontario and McMaster Universities Osteoarthritis Index) and KOOS (Knee injury and Osteoarthritis Outcome Score) subgroups

Significant changes in the primary pain outcomes were observed across all time points in the overall cohort **(**Fig. 3; Table 3; Supplementary Table 2). Mean VAS improved from 8.13 (95% CI: 7.76, 8.51) at baseline to 2.74 (95% CI: 2.17, 3.30) at 30 days, 3.35 (95% CI: 2.67, 4.03) at 90 days, and 3.92 (95% CI: 3.20, 4.63) at 365 days (all p < 0.001). Mean WOMAC pain improved from 13.93 (95% CI: 13.23, 14.64) to 5.52 (95% CI: 4.47, 6.56), 7.02 (95% CI: 5.92, 8.12), and 6.91 (95% CI: 5.68, 8.14), respectively (all p < 0.001). Mean KOOS pain improved from 30.64 (95% CI: 27.14, 34.14) to 68.00 (95% CI: 62.59, 73.41), 62.05 (95% CI: 56.11, 67.99), and 59.56 (95% CI: 52.64, 66.49), respectively (all p < 0.001).

**Table 3:** Changes in VAS, WOMAC Pain, and KOOS Pain over the one-year follow-up period

	Global				*I*/*C*				Microspheres			
	Baseline	30 days	90 days	365 days	Baseline	30 days	90 days	365 days	Baseline	30 days	90 days	365 days
VAS	8.13 (95% CI: 7.76, 8.51)	2.74 (95% CI: 2.17, 3.30)	3.35 (95% CI: 2.67, 4.03)	3.92 (95% CI: 3.20, 4.63)	7.93 (95% CI: 7.41, 8.44)	3.2 (95% CI: 2.3, 4.1)	3.38 (95% CI: 2.51, 4.26)	3.95 (95% CI: 3.04, 4.85)	8.33 (95% CI: 7.79, 8.88)	2.28 (95% CI: 1.63, 2.92)	3.33 (95% CI: 2.29, 4.36)	3.88 (95% CI: 2.76, 5.01)
*p* value		< 0.001	< 0.001	< 0.001		< 0.001	< 0.001	< 0.001		< 0.001	< 0.001	< 0.001
WOMAC Pain	13.93 (95% CI: 13.23, 14.64)	5.52 (95% CI: 4.47, 6.56)	7.02 (95% CI: 5.92, 8.12)	6.91 (95% CI: 5.68, 8.14)	14.17 (95% CI: 13.38, 14.95)	6.25 (95% CI: 4.58, 7.92)	8.11 (95% CI: 6.66, 9.56)	7.5 (95% CI: 5.85, 9.15)	13.7 (95% CI: 12.54, 14.86)	4.83 (95% CI: 3.59, 6.08)	6.0 (95% CI: 4.45, 7.55)	6.27 (95% CI: 4.47, 8.06)
*n* (%), *N*	60 (100%), 60	30 (51.72%), 58	27 (48.2%), 56	30 (55.5%), 54	30 (100%), 30	19 (67.9%), 28	10 (37%), 27	10 (41.7%), 28	30 (100%), 30	19 (70.4%), 30	16 (57.1%), 29	13 (61.9%), 26
*p* value		< 0.001	< 0.001	< 0.001		< 0.001	< 0.001	< 0.001		< 0.001	< 0.001	< 0.001
KOOS Pain	30.64 (95% CI: 27.14, 34.14)	68 (95% CI: 62.59, 73.41)	62.05 (95% CI: 56.11, 67.99)	59.56 (95% CI: 52.64, 66.49)	29.53 (95% CI: 25.53, 33.91)	63.59 (95% CI: 55.62, 71.55)	56.58 (95% CI: 48.24, 64.92)	53.76 (95% CI: 44.91, 62.62)	31.75 (95% CI: 26.32, 37.19)	72.12 (95% CI: 65.08, 79.16)	67.14 (95% CI: 59.13, 75.15)	65.81 (95% CI: 55.56, 76.06)
*n* (%), *N*	60 (100%), 60	51 (87.9%), 58	47 (83.9%), 56	42 (77.7%), 54	30 (100%), 30	22 (78.5%), 28	20 (74.0%), 27	17 (60.7%), 28	30 (100%), 30	25 (83.3%), 30	24 (82.7%), 29	16 (61.5%), 26
*p* value		< 0.001	< 0.001	< 0.001		< 0.001	< 0.001	< 0.001		< 0.001	< 0.001	< 0.001

Legend: *I*/*C* = imipenem/cilastatin; *n* = patients who reached the endpoint; *N* = total number of patients evaluated at that time

Similar improvements in the primary pain outcomes were observed in both groups. In the *I*/*C* group, mean VAS improved from 7.93 (95% CI: 7.41, 8.44) at baseline to 3.20 (95% CI: 2.30, 4.10) at 30 days, 3.38 (95% CI: 2.51, 4.26) at 90 days, and 3.95 (95% CI: 3.04, 4.85) at 365 days. In the permanent microsphere group, mean VAS improved from 8.33 (95% CI: 7.79, 8.88) to 2.28 (95% CI: 1.63, 2.92), 3.33 (95% CI: 2.29, 4.36), and 3.88 (95% CI: 2.76, 5.01), respectively. Mean WOMAC pain improved from 14.17 (95% CI: 13.38, 14.95) to 6.57 (95% CI: 5.11, 8.04), 7.47 (95% CI: 6.08, 8.87), and 7.50 (95% CI: 5.85, 9.15) in the *I*/*C* group, and from 13.70 (95% CI: 12.54, 14.86) to 4.46 (95% CI: 2.98, 5.94), 6.57 (95% CI: 4.90, 8.24), and 6.27 (95% CI: 4.47, 8.06) in the permanent microsphere group. Mean KOOS pain improved from 29.53 (95% CI: 25.15, 33.91) to 61.40 (95% CI: 53.09, 69.71), 56.94 (95% CI: 48.00, 65.89), and 53.76 (95% CI: 44.91, 62.62) in the *I*/*C* group and from 31.75 (95% CI: 26.32, 37.19) to 74.59 (95% CI: 66.83, 82.34), 67.16 (95% CI: 57.84, 76.48), and 65.81 (95% CI: 55.56, 76.06) in the permanent microsphere group.

When KOOS outcomes were compared between groups, significant differences were observed for the KOOS symptoms (*p* = 0.009) and sports (*p* = 0.025) subscales at 30 days and for the KOOS quality of life subscale at 30 and 90 days (*p* = 0.048 and *p* = 0.05, respectively) in favor of PM. These differences were not maintained at 365 days.

### Safety

Six patients were lost to follow-up (*I/C* *n* = 2; PM *n* = 4), including one patient who died unrelated to embolization 7 months post-procedure. The death was due to respiratory complications secondary to a COVID-19 infection.

**Fig. 4:**
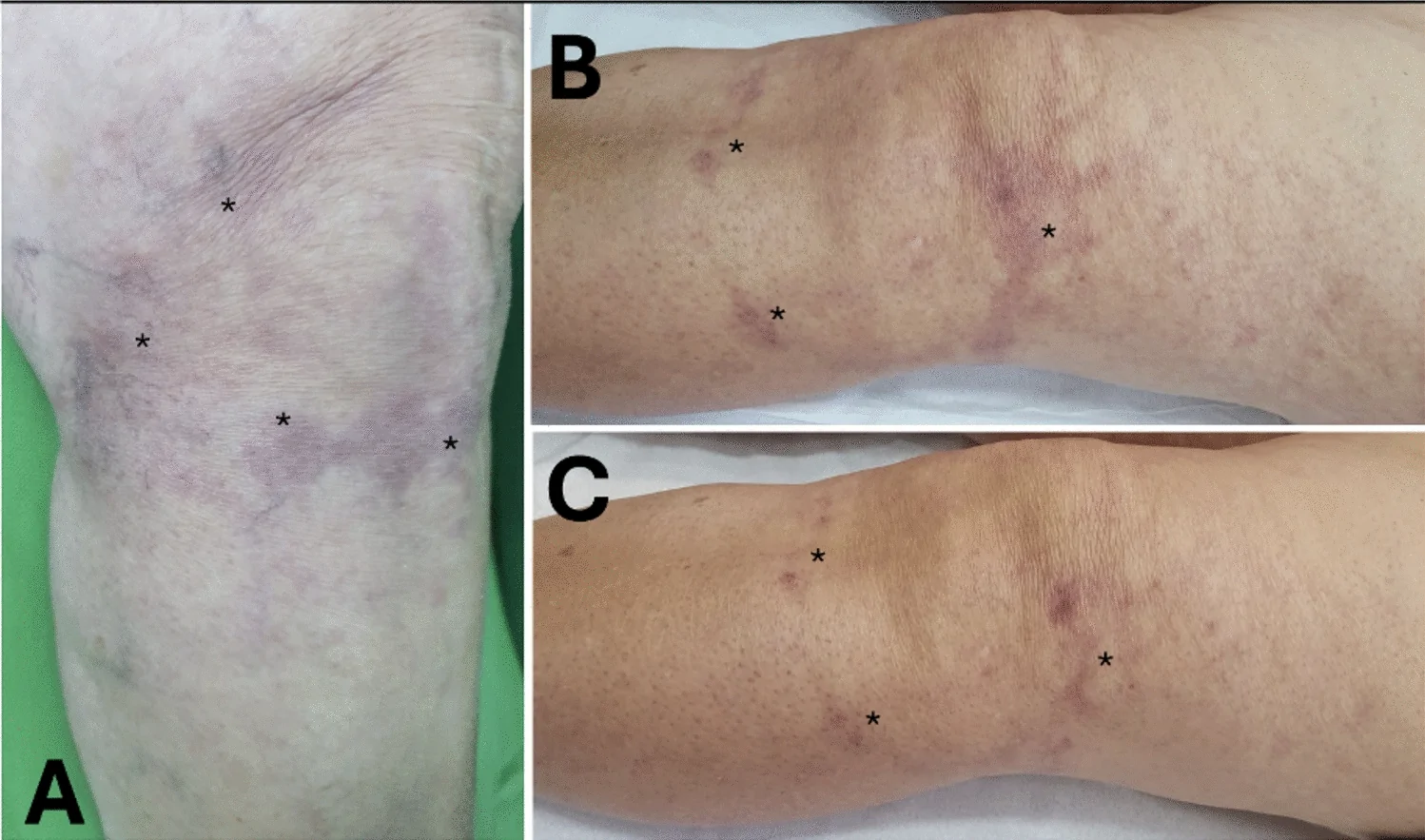
Skin discoloration (*) on the anterior aspect of a right (**A**) and left (**B**) knee immediately after the procedure. After a six-hour rest period, there is almost complete remission of livedo (**C**) in the patient in image **B**, which disappeared defi nitively over the next hours

Seven minor procedure-related complications occurred. The most frequent adverse event was temporary skin discoloration (SD), which occurred in 39 knees (47.6%), one of which was painful. It occurred in 19 knees (45.2%) in the *I*/*C* and in 20 (50%) knees in the PM group. There was no significant difference between groups (*p* = 0.907). Most patients had spontaneous resolution in the first hours after the procedure (Fig. 4). One SD in the PM group was painful and resolved spontaneously within 24h. One prolonged SD in the PM group resolved after 180 days, with only cosmetic complaints and empiric use of aspirin 100 mg and rivaroxaban 20 mg for 30 days. There were no clinical or MRI signs of major complications (e.g., osteonecrosis, non-target embolization, ischemia) (Table 4).

**Table 4:** Adverse events and complications

Adverse events and complications	Type	Treatment	*I*/*C*	Microsphere	*P* value
Temporary skin discoloration	Minor	observation	19	20	0.665
Access hematoma	Minor	observation	2	2	1
Local skin edema	Minor	observation	2	0	0.489

*N*—number of knees; % percentage; *I*/*C*—imipenem/cilastatin

### Medications

There was a significant reduction in the use of all classes of medications after GAE, particularly opioids, that persisted over the one-year follow-up period (*p* < 0.001). No significant difference in medication use between the two treatment groups was observed at any time during the follow-up period (Table 5).

**Table 5:** Medications

	*I*/*C*		Microspheres		*P* value
NSAIDS	Baseline	20	Baseline	27	0.092
	30 days	5	30 days	4	1.0
	90 days	4	90 days	5	1.0
	365 days	4	365 days	7	0.501
*p* value	Baseline—365 days	< 0.001	Baseline—365 days	< 0.001	
Analgesics	Baseline	24	Baseline	22	0.576
	30 days	12	30 days	5	0.097
	90 days	10	90 days	9	1.0
	365 days	6	365 days	6	1.0
*p* value	Baseline—365 days	< 0.001	Baseline—365 days	< 0.001	
Opioids	Baseline	11	Baseline	6	0.249
	30 days	1	30 days	3	0.577
	90 days	3	90 days	2	1.0
	365 days	0	365 days	1	1.0
*p* value	Baseline—365 days	< 0.001	Baseline—365 days	< 0.001	
Neuropathic	Baseline	5	Baseline	2	0.394
	30 days	3	30 days	0	0.235
	90 days	3	90 days	0	0.236
	365 days	1	365 days	2	1.0
*p* value	Baseline—365 days	< 0.001	Baseline—365 days	< 0.001	

Legend: *I*/*C* = imipenem/cilastatin; NSAID—Non-steroid anti-inflammatory drugs

### Sub-analysis

A separate sub-analysis was performed according to disease severity, comparing patients with mild osteoarthritis (KL 1–2) and moderate osteoarthritis (KL 3). Significant differences were observed at 90 days for WOMAC activities of daily living (*p* = 0.038) and KOOS quality of life (*p* = 0.010), favoring patients with less advanced disease. However, these differences were not maintained at 365 days (Supplemental Table 3).

## Discussion

Results from this trial demonstrate that GAE with* I*/*C* or permanent microspheres (100–300 μm) is safe and effective, with no major adverse events observed over the 12-month follow-up period. The main finding of this study is that both embolic agents achieved similar clinical improvement in the main pain outcomes over the follow-up, with only transient differences in selected KOOS subscales at certain follow-up times. This may have been due to the difference in bilaterality between the groups. However, since the questionnaires are completed per patient, not per knee, these slight changes are not sustained in the long term. Given the limited comparative literature on embolic agents in GAE, this trial addresses an important gap in the current evidence.

Reduction in pain scores was the primary outcome used to assess efficacy and was similar in both groups. In this study, both embolic agents effectively improved symptoms, joint functionality, and patients’ quality of life. Overall, the results from this trial are consistent with the findings from prior cohort studies demonstrating that both embolic agents are effective in improving symptoms, function after GAE [9, 19]. More recent comparative data also support this interpretation. In a retrospective study comparing lipiodol versus imipenem/cilastatin, Guzelbey et al. reported similar clinical success and safety profiles between two temporary embolic strategies, with only limited differences in specific WOMAC domains [20]. In another comparative study, Badar et al. reported that temporary ethiodized oil emulsion and permanent polyethylene glycol microspheres showed differences in short-term pain response, but these differences were less pronounced at mid-term follow-up, again suggesting that embolic type may influence early response more than overall durability [21]. A recent comprehensive review also concluded that both temporary and permanent embolic agents appear to be effective, although heterogeneity in patient selection, embolic technique, and endpoint definition still limits direct comparison across studies [22].

When interpreting the clinical scores, WOMAC and KOOS provide complementary information. WOMAC is more directly focused on pain, stiffness, and physical limitation in osteoarthritis, whereas KOOS captures additional dimensions such as symptoms, sports/recreation, and knee-related quality of life. This may explain why differences between embolic agents in this study were observed only in selected KOOS subdomains, particularly symptoms, sports, and quality of life, and mainly at early follow-up. These findings should be interpreted with caution, since they were secondary outcomes and were not consistently sustained at 12 months.

The definition of success in reducing pain using WOMAC and KOOS often involves the concepts of minimal clinically important difference (MCID) and Patient Assessment Symptoms State (PASS), which represent thresholds of clinically meaningful improvement and acceptable symptom control. The minimum relevant efficacy thresholds reported in knee osteoarthritis are approximately 20% [23, 24]. In this trial, there was a reduction of 7.02 points in the WOMAC pain averages at 12 months, corresponding to a reduction of 49.5%. This is twice the estimated value for the variable. PASS has been reported as 82.8 for the total WOMAC score [25]. In this trial, all patients achieved this result.

Regarding the KOOS, the MCID for the pain subdomain in patients undergoing total knee arthroplasty has been reported as 7.9, with a PASS threshold of 77.7 [26]. Therefore, an improvement of 7.9% can be considered clinically meaningful. In this study, a 10% increase was used as the criterion for clinical success and was found in 77.7% of patients in 12 months, with no difference between embolic agents.

The Reporting Standards from the Society of Interventional Radiology define clinical success for GAE as a 20% reduction in WOMAC pain [27]. However, there is still heterogeneity among published studies. For this trial, a 50% reduction in WOMAC was used to define clinical success. This reinforces that, despite variability in endpoint definition, the magnitude of pain reduction observed in this study was clinically relevant.

Although no major adverse events occurred in this study, SD was observed in 47.6% of cases, with one case associated with pain. The relatively high rate observed in this study may be related to the absence of a standardized definition or classification of SD. In this study, any skin alteration, however subtle, was considered SD. Despite a high rate of skin discoloration, there was no difference between treatment groups. Most published cohorts report variable rates of skin discoloration [28–32]. To reduce the risk of skin discoloration, some groups are using ice packs for 15 min prior to embolization to produce cutaneous vasoconstriction; however, the occurrence of discoloration varies from 10 to 65% [5–8, 32, 33]. In this trial, ice packs were not used to avoid a confounding factor and to document the true incidence in each group. It is possible that this strategy could have reduced the frequency of this event. Skin discoloration, although prevalent, was self-limited in most patients, mostly painless, with resolution within the first few hours after treatment, while still resting in the recovery unit, and was considered a minor adverse event in both SIR and CIRSE classifications [18, 34]. Importantly, there were no clinical or MRI signs of major ischemic complications.

This point is relevant when comparing temporary and permanent embolic agents, since concern regarding prolonged ischemia has historically favored the use of temporary embolization strategies. Rouzbahani et al. recently reviewed the use of imipenem/cilastatin in embolization and emphasized its transient embolic behavior and potential theoretical advantage in reducing prolonged ischemic injury, while also discussing the practical and regulatory drawbacks of using an antibiotic as an embolic agent [2]. In parallel, Badar et al. observed fewer overall adverse events and cutaneous changes with a temporary embolic strategy compared with permanent microspheres, suggesting that the safety profile of the embolic agent remains an important technical consideration in GAE [21].

In this trial, a sub-analysis according to disease severity showed significant differences at 90 days for WOMAC activities of daily living and KOOS quality of life, favoring patients with less advanced disease (KL 1–2). However, these differences were not maintained at 365 days. These findings suggest that baseline disease severity may influence early response to GAE and should be considered during patient selection and counseling [28, 30, 35].

Overall, there was a significant reduction in the use of analgesics, NSAIDs, opioids, and medications for neuropathic pain throughout the follow-up. No difference between embolic agents was observed in this respect.

There are key technical details of this study that should be noted. First, procedures were performed under local anesthesia and without sedation. This allowed patients to confirm that the appropriate artery was targeted and may have contributed to the reduction in procedure time, an improved immediate clinical outcome, and may have reduced the number of arteries treated while maintaining similar clinical results. A recent study suggested that patients who reported symptoms during injection (evoked pain) achieved clinical results more frequently than those who did not.^7^ In this context, “*clinically driven embolization*” may help reduce unnecessary embolization and potentially limit ischemic events related to larger embolic volumes. However, it should be interpreted with caution, since it may not be easily reproducible in all patients or centers, particularly in patients who do not report clear symptoms during the procedure or whose pain is difficult to localize in the supine position. In addition, its role needs further validation. Second, the use of an IM catheter for catheterization of the ostia of the target arteries, may reduce the time of the procedure, reducing radiation exposure. Third, injecting always 0.3-mL aliquots of embolic agent with a 1-mL syringe as long as there was blush allowed both embolic agents to be delivered in a similar stepwise manner.

While this study addresses an important gap in the literature to date, it has limitations. It was conducted at a single center; the operators were not blinded to the embolic agent and there was no non-GAE comparator. Another limitation is the unequal distribution of bilateral GAE between groups, which may have influenced the initial comparative outcomes, although this effect was not maintained at the end of follow-up. In addition, early post-procedural pain was not systematically assessed between discharge and the 30-day visit, which limits interpretation of initial pain.

## Conclusion

GAE using either *I*/*C* or permanent microspheres was safe and effective in patients with symptomatic knee osteoarthritis. Clinical outcomes were similar between embolic agents over 12 months, although selected KOOS subscales showed transient early differences that were not maintained throughout follow-up. These findings suggest that both *I*/*C* and permanent microspheres are feasible embolic agents for GAE.

## Supplementary Information

Below is the link to the electronic supplementary material.

Supplementary file1 (DOCX 16 KB)

Supplementary file2 (DOCX 24 KB)

Supplementary file3 (DOCX 20 KB)

## Data Availability

Not applicable.
