# Correction to: Genicular Artery Embolization Using Imipenem/Cilastatin vs. Microsphere for Knee Osteoarthritis (GAUCHO). 12-Month Clinical Results of a Single-blind Randomized Controlled Trial

**DOI:** 10.1007/s00270-026-04583-4

**Published:** 2026-08-27

**Authors:** Mateus Picada Correa, Alexandre Froes Michelin, Lyson Azevedo Aguiar, Ricardo Lugokenski, Rodrigo Ilha Algarve, Joaquim M. Motta‑Leal‑Filho

**Affiliations:** 1 Instituto Vascular de Passo Fundo (Invasc), R. Capitão Araújo 297/1207, Passo Fundo, RS 99010-200 Brazil; 2 Universidade de Passo Fundo (UPF), R. Uruguai, 2001/414B, Passo Fundo, RS 99010-112 Brazil; 3 Atitus Educação, R. Gen Prestes Guimarães, 304, Passo Fundo, RS 999070-220 Brazil; 4 Hospital Ortopédico de Passo Fundo, Av. 7 de Setembro, 817, Passo Fundo, Brazil; 5 Clínica Cedil, Av. 7 de Setembro, 817, Passo Fundo, Brazil; 6 School of Medicine, Instituto Do Câncer Do Estado de São Paulo, Universidade de São Paulo (USP), Av. Dr. Arnaldo, 251, Cerqueira Cesar, São Paulo, SP 01246-000 Brazil; 7 School of Medicine, Instituto Do Coração (InCor), Universidade de São Paulo (USP), Av. Dr. Enéas de Carvalho Aguiar, 44, São Paulo, SP 05403-900 Brazil

**Correction to: Cardiovasc Intervent Radiol** https://doi.org/10.1007/s00270-026-04504-5

In the original publication of this article, the institution name in the Study Design section was published incorrectly.

Incorrect:

“This was a randomized, single-blind, prospective, single-center trial approved by the Ethics Committee of the University of XXXXXX.”

Correct:

“This was a randomized, single-blind, prospective, single-center trial approved by the Ethics Committee of Universidade de Passo Fundo.”

The original article has been corrected.

