# Supplementary material for: Genicular Artery Embolization Using Imipenem/Cilastatin vs. Microsphere for Knee Osteoarthritis (GAUCHO). 12-Month Clinical Results of a Single-blind Randomized Controlled Trial
Source: Cardiovasc Intervent Radiol. 2026 Jun 29;49(9):1825–37. doi: 10.1007/s00270-026-04504-5 (PMC13597651; doi:10.1007/s00270-026-04504-5)
Supplement: Supplementary file 1 [file 270_2026_4504_MOESM1_ESM.docx]

| **Inclusion Criteria** | **Exclusion Criteria** |
| --- | --- |
| 1. Diagnosis of knee osteoarthritis: Kellgren-Lawrence grades 1-3 confirmed on X-ray. 2. At least six months of knee pain related to osteoarthritis. 3. Refractory to at least six months of conservative management (i.e., physical therapy, parenteral medications, intra-articular corticosteroid infiltration) without clinical improvement. 4. Synovitis in the knee confirmed by magnetic resonance imaging. 5. Sign the informed consent form and agree to participate in clinical and radiological follow-up. | 1. Candidate for knee arthroplasty (i.e. Kellgren-Lawrence grade 4); 2. Underwent previous total knee replacement surgery. 3. Diagnosis of fibromyalgia, rheumatoid arthritis or infectious arthritis. 4. Use of long-term intramuscular steroids in the 30 days preceding the GAE procedure. 5. Being unable to accurately describe areas of pain during the procedure or refusing to participate in the study protocol. 6. Allergic to iodinated contrast or to any of the embolic agents. 7. Severe coagulopathies; 8. Prothrombin activity time < 50%; 9. INR > 1.5; 10. Platelets < 50.000 por mm^3^; 11. Serum Creatinine > 2.0mg/dl. |

GAE = genicular artery embolization, INR = international normalized ratio.
