# Supplementary material for: Genicular Artery Embolization Using Imipenem/Cilastatin vs. Microsphere for Knee Osteoarthritis (GAUCHO). 12-Month Clinical Results of a Single-blind Randomized Controlled Trial
Source: Cardiovasc Intervent Radiol. 2026 Jun 29;49(9):1825–37. doi: 10.1007/s00270-026-04504-5 (PMC13597651; doi:10.1007/s00270-026-04504-5)
Supplement: Supplementary file 2 [file 270_2026_4504_MOESM2_ESM.docx]

Supplemental Table 2: WOMAC Total, Stiffness and Activities.

|  | **Global** | | | | **I/C** | | | | **Microspheres** | | | |
| --- | --- | --- | --- | --- | --- | --- | --- | --- | --- | --- | --- | --- |
|  | **Baseline** | **30 days** | **90 days** | **365 days** | **Baseline** | **30 days** | **90 days** | **365 days** | **Baseline** | **30 days** | **90 days** | **365 days** |
| **WOMAC Total** | **68.18**  (95% CI: 65.06, 71.30) | **28.36**  (95% CI: 23.27, 33.46) | **34.34**  (95% CI: 29.38, 39.30) | **35.24**  (95% CI: 29.39, 41.09) | **67.7**  (95% CI: 64.08, 71.32) | **32.96**  (95% CI: 25, 40.93) | **38.96**  (95% CI: 32.21, 45.72) | **38.46**  (95% CI: 30.73, 46.19) | **68.67**  (95% CI: 63.54, 73.74) | **24.07**  (95% CI: 18, 30.13) | **30.03**  (95% CI: 23.18, 36.89) | **31.77**  (95% CI: 23.11, 40.43) |
| **N** | 60 | 58 | 56 | 54 | 30 | 28 | 27 | 28 | 30 | 30 | 29 | 26 |
| **p-value** |  | <0.001 | <0.001 | <0.001 |  | <0.001 | <0.001 | <0.001 |  | <0.001 | <0.001 | <0.001 |
|  |  |  |  |  |  |  |  |  |  |  |  |  |
| **WOMAC Stiffness** | **5.48**  (95% CI: 5.10, 5.87) | **2.67**  (95% CI: 2.15, 3.19) | **2.98**  (95% CI: 2.5, 3.46) | **3.24**  (95% CI: 2.67, 3.81) | **5.33**  (95% CI: 4.83, 5.83) | **2.89**  (95% CI: 2.14, 3.65) | **3.22**  (95% CI: 2.54, 3.91) | **3.61**  (95% CI: 2.75, 4.47) | **5.63**  (95% CI: 5.05, 6.21) | **2.47**  (95% CI: 1.76, 3.17) | **2.76**  (95% CI: 2.10, 3.42) | **2.85**  (95% CI: 2.13,3.57) |
| **N** | 60 | 58 | 56 | 54 | 30 | 28 | 27 | 28 | 30 | 30 | 29 | 26 |
| **p-value** |  | <0.001 | <0.001 | <0.001 |  | <0.001 | <0.001 | <0.001 |  | <0.001 | <0.001 | <0.001 |
|  |  |  |  |  |  |  |  |  |  |  |  |  |
| **WOMAC Activities** | **48.70**  (95% CI: 48.39, 51.01) | **20.17**  (95% CI: 16.43, 23.91) | **24.39**  (95% CI: 20.69, 28.09) | **25.09**  (95% CI: 20.85, 29.34) | **48.20**  (95% CI: 45.43, 50.97) | **23.82**  (95% CI: 17.92, 29.72) | **27.63**  (95% CI: 22.63, 32.63) | **27.36**  (95% CI: 21.78, 32.93) | **49.2**  (95% CI: 45.5, 52.9) | **16.77**  (95% CI: 12.41, 21.12) | **21.38**  (95% CI: 16.19, 26.57) | **22.65**  (95% CI: 16.33, 28.97) |
| **N** | 60 | 58 | 56 | 54 | 30 | 28 | 27 | 28 | 30 | 30 | 29 | 26 |
| **p-value** |  | <0.001 | <0.001 | <0.001 |  | <0.001 | <0.001 | <0.001 |  | <0.001 | <0.001 | <0.001 |
| **KOOS Total** | **27.43**  (95% CI: 24.57, 30.29) | **61.36**  (95% CI: 56.12, 66.59) | **56.35**  (95% CI: 50.83, 61.87) | **53.81**  (95% CI: 47.55, 60.07) | **26.66**  (95% CI: 23.28, 30.05) | **55.90**  (95% CI: 48.47, 63.34) | **50.99**  (95% CI: 43.41, 58.57) | **59.68**  (95% CI: 53.90, 68.89) | **28.19**  (95% CI: 23.59, 32.78) | **66.44**  (95% CI: 59.57, 73.32) | **61.35**  (95% CI: 53.90, 68.89) | **59.68**  (95% CI: 50.74, 68.62) |
| **N** | 60 | 58 | 56 | 54 | 30 | 28 | 27 | 28 | 30 | 30 | 29 | 26 |
| **p-value** |  | <0.001 | <0.001 | <0.001 |  | <0.001 | <0.001 | <0.001 |  | <0.001 | <0.001 | <0.001 |
| **KOOS Symptoms** | **36.54**  (95% CI: 32.90, 40.18) | **64.40**  (95% CI: 59.20, 69.60) | **59.24**  (95% CI: 53.46, 65.03) | **55.09**  (95% CI: 48.46, 61.72) | **36.90**  (95% CI: 32.70, 41.10) | **57.65**  (95% CI: 51.08, 64.21) | **53.96**  (95% CI: 46.02, 61.91) | **51.27**  (95% CI: 43.05, 59.49) | **36.19**  (95% CI: 30.25, 42.12) | **70.71**  (95% CI: 63.43, 77.99) | **64.16**  (95% CI: 56.19, 72.13) | **59.20**  (95% CI: 48. 89,69. 51) |
| **N** | 60 | 58 | 56 | 54 | 30 | 28 | 27 | 28 | 30 | 30 | 29 | 26 |
| **p-value** |  | <0.001 | <0.001 | <0.001 |  | <0.001 | <0.001 | <0.001 |  | <0.001 | <0.001 | <0.001 |
|  |  |  |  |  |  |  |  |  |  |  |  |  |
| **KOOS Activities** | **30.04**  (95% CI: 26.18, 39.0) | **68.30**  (95% CI: 62.50, 74.10) | **62.65**  (95% CI: 57.14, 68.17) | **60.64**  (95% CI: 54.13, 67.16) | **29.90**  (95% CI: 24.89, 34.90) | **64.23**  (95% CI: 55.41, 73.05) | **57.46**  (95% CI: 49.50, 65.42) | **55.56**  (95% CI: 46.26, 64.87) | **30.19**  (95% CI: 24.31, 36.07) | **72.10**  (95% CI: 64.75, 79.05) | **67.49**  (95% CI: 60.28, 74.70) | **66.11**  (95% CI: 57.50, 74.73) |
| **N** | 60 | 58 | 56 | 54 | 30 | 28 | 27 | 28 | 30 | 30 | 29 | 26 |
| **p-value** |  | <0.001 | <0.001 | <0.001 |  | <0.001 | <0.001 | <0.001 |  | <0.001 | <0.001 | <0.001 |
| **KOOS**  **Sports** | **7.41**  (95% CI: 4.58, 10.25) | **34.05**  (95% CI: 25.94, 42.16) | **32.41**  (95% CI: 24.65, 40.16) | **28.51**  (95% CI: 20.27, 36.75) | **4.33**  (95% CI: 2.13, 6.53) | **24.82**  (95% CI: 13.54, 36.10) | **27.03**  (95% CI: 17.76, 36.30) | **20.89**  (95% CI: 10.91, 30.86) | **10.50**  (95% CI: 5.51, 15.48) | **42.66**  (95% CI: 31.93, 53.39) | **37.41**  (95% CI: 25.46, 49.36) | **36.73**  (95% CI: 24.15, 49.31) |
| **N** | 60 | 58 | 56 | 54 | 30 | 28 | 27 | 28 | 30 | 30 | 29 | 26 |
| **p-value** |  | <0.001 | <0.001 | <0.001 |  | <0.002 | <0.001 | <0.008 |  | <0.001 | <0.001 | <0.001 |
| **KOOS**  **QoL** | **18.12**  (95% CI: 15.31, 20.93) | **45.68**  (95% CI: 39.09, 52.28) | **41.40**  (95% CI: 35.15, 47.65) | **41.66**  (95% CI: 34.89, 48.43) | **16.45**  (95% CI: 12.65, 20.26) | **39.06**  (95% CI: 30.40, 47.71) | **35.18**  (95% CI: 27.80, 42.56) | **35.71**  (95% CI: 27.80, 42.56) | **19.79**  (95% CI: 15.74, 23.83) | **51.87**  (95% CI: 42.54, 61.20) | **47.19**  (95% CI: 37.74, 56.65) | **48.07**  (95% CI: 38.17, 57.97) |
| **N** | 60 | 58 | 56 | 54 | 30 | 28 | 27 | 28 | 30 | 30 | 29 | 26 |
| **p-value** |  | <0.001 | <0.001 | <0.001 |  | <0.001 | <0.001 | <0.001 |  | <0.001 | <0.001 | <0.001 |

Legend: I/C = Imipenem/Cilastatin; n = patients who reached the endpoint; N = total number of patients evaluated at that time
