# Supplementary material for: Genicular Artery Embolization Using Imipenem/Cilastatin vs. Microsphere for Knee Osteoarthritis (GAUCHO). 12-Month Clinical Results of a Single-blind Randomized Controlled Trial
Source: Cardiovasc Intervent Radiol. 2026 Jun 29;49(9):1825–37. doi: 10.1007/s00270-026-04504-5 (PMC13597651; doi:10.1007/s00270-026-04504-5)
Supplement: Supplementary file 3 [file 270_2026_4504_MOESM3_ESM.docx]

**Supplementary Table 3:** Kellgren-Lawrence sub analysis

|  |  | **pre** | **30 days** | **90 days** | **365 days** |
| --- | --- | --- | --- | --- | --- |
| **VAS** | KL 1-2 | 8.00 | 2.71 | 3.16 | 3.60 |
|  | KL 3 | 8.58 | 2.82 | 4.06 | 5.06 |
|  | ***p*** | ***0.171*** | ***0.860*** | ***0.301*** | ***0.142*** |
| **WOMAC**  **pain** | KL 1-2 | 13.85 | 5.48 | 6.53 | 6.29 |
|  | KL 3 | 14.21 | 5.67 | 8.62 | 9.08 |
|  | ***p*** | ***0.662*** | ***0.863*** | ***0.106*** | ***0.077*** |
| **WOMAC rigity** | KL 1-2 | 5.48 | 2.59 | 2.86 | 3.05 |
|  | KL 3 | 5.50 | 3.00 | 3.38 | 3.92 |
|  | ***p*** | *0.960* | *0.542* | *0.329* | *0.195* |
| **WOMAC**  **Qol** | KL 1-2 | 48.28 | 19.00 | 22.23 | 23.14 |
|  | KL 3 | 50.07 | 24.67 | 31.54 | 31.92 |
|  | ***p*** | ***0.441*** | ***0.185*** | ***0.038*** | ***0.101*** |
| **WOMAC total** | KL 1-2 | 67.61 | 27.07 | 31.63 | 32.48 |
|  | KL 3 | 70.07 | 33.33 | 43.31 | 44.92 |
|  | ***p*** | ***0.433*** | ***0.284*** | ***0.048*** | ***0.093*** |
| **KOOS pain** | KL 1-2 | 31.70 | 69.32 | 64.79 | 63.16 |
|  | KL 3 | 27.18 | 62.96 | 52.99 | 46.99 |
|  | ***p*** | ***0.261*** | ***0.366*** | ***0.113*** | ***0.069*** |
| **KOOS symptoms** | KL 1-2 | 36.49 | 64.9 | 61.96 | 57.39 |
|  | KL 3 | 36.73 | 62.5 | 50.27 | 47.02 |
|  | ***p*** | ***0.952*** | ***0.732*** | ***0.128*** | ***0.242*** |
| **KOOS ADL** | KL 1-2 | 32.22 | 69.59 | 65.28 | 63.97 |
|  | KL 3 | 22.89 | 63.35 | 53.95 | 49.01 |
|  | ***p*** | ***0.19*** | ***0.372*** | ***0.103*** | ***0.063*** |
| **KOOS sports** | KL 1-2 | 8.15 | 35.00 | 35.81 | 31.30 |
|  | KL 3 | 5.00 | 30.41 | 21.15 | 18.75 |
|  | ***p*** | ***0.209*** | ***0.655*** | ***0.107*** | ***0.149*** |
| **KOOS QoL** | KL 1-2 | 19.02 | 48.36 | 45.34 | 42.85 |
|  | KL 3 | 15.17 | 35.41 | 28.36 | 37.50 |
|  | ***p*** | ***0.203*** | ***0.094*** | ***0.010*** | ***0.448*** |
| **KOOS total** | KL 1-2 | 28.70 | 62.61 | 59.24 | 56.74 |
|  | KL 3 | 23.25 | 56.54 | 46.79 | 43.55 |
|  | ***p*** | ***0.078*** | ***0.369*** | ***0.073*** | ***0.089*** |

VAS – Visual Analogue Scale of Pain, KOOS= Knee Injury and Osteoarthritis Outcome Score; WOMAC= Western Ontario and McMaster Universities Osteoarthritis Index; QoL = Quality of life; ADL = Activities of daily living; KL – Kellgren-Lawrence.
